# Women Physicians at Clapham

**Published:** 1889-09-28

**Authors:** 


### Women Physicians at Clapham.

*(By a Clerical Correspondent.)*

Readers of The Hospital already know something of the Clapham School of Midwifery, begun some four years ago by Dr. Annie McCall. In this period some twelve hundred cases have been attended without a single death. This out-patient maternity work having been so successful, one is not surprised to find that it was decided to open a small lying-in hospital (twelve beds), mainly for the respectable married poor of this populous district of Clapham, where a suitable home has been obtained for the Clapham Maternity Hospital, opened last May. The hospital is within a few minutes' walk of the General Dispensary for Women and Children, where out-patients are seen by women physicians three times a week. At this dispensary we notice there were 9,600 attendances last year.

Our present purpose being to draw attention to the principles upon which this twofold work is carried forward, we need not further distinguish between the dispensary and the hospital, the same principles ruling in each case and being identical so far as the work allows. We have personally visited both, and were most courteously shown the whole working of the two places by Dr. McCall and the hon. secretary, Mrs. Ritchie. The dispensary is at 131, Clapham-road, and the hospital—a wholesome, pleasant home in a good position—is at 74, Jeffreys-road. Any of the patients who could be seen were comfortable and composed, evidently doing well, both themselves and their children receiving the utmost and kindliest care and attention. The whole house was remarkably clean, well ordered, ventilated, and, as we have said, wholesome.

Primarily, this maternity work at Clapham is an appeal to those amongst the poor who desire to be attended by women physicians. It is simply beyond the smallest doubt that there has been a wonderful response to this appeal, a response which will surprise some of us, and which must be particularly gratifying and encouraging to Dr. McCall, whose soul is surely in this work. Need we say that we mean no disrespect to women physicians in acknowledging some surprise at their success amongst the poor. We are remembering that none are more deeply rooted in old habits and prejudices than the poor, and to have brought them to anything so novel as attendance from "lady doctors" (as we say) is undoubtedly a great success. True, many poor women do not count upon medical attendance in ordinary cases of maternity. But those who do not are the very poor; they are not that class who pay 5s. a week whilst lying-in, or pay at the dispensary for advice there and attendance at their own homes. This is the class from which the Clapham School of Midwifery recruits its clients.

There were also other obstacles. Is there anyone in the profession who has not heard women say, over and over again, that nothing would induce them to go under the care of women physicians? Qualified women will say, of course, that this feeling is entirely due to the novelty of women physicians. Well, not entirely, though perhaps largely. There may be those who regret it, but it does seem simply human nature for woman to "put confidence in man." Even should this confidence be sometimes misplaced, the feeling itself will never be eradicated. It is a disadvantage that women physicians will always have to reckon with, one, indeed, of many. But all difficulties seem to disappear at Clapham. The women have been found who prefer women physicians, and the record of success fairly guarantees the clients of the future. A lot of people think the age of superstition is past and gone, and would be surprised to hear that the London poor like to get married in "a lucky church." The demands that would be made on a lucky lying-in hospital we really must not speculate upon.

The training giving to young medical women is another very special feature of the work. In a circular issued in connection with the opening of the hospital we find the following: "This is, we believe, the only institution where women can receive instruction in practical midwifery as medical students, and not merely as midwives. It is therefore desired to render it more complete by the addition of in-patients, as the clinical teaching can be more thorough than in out-patient practice, and experience in the management of in-patients can be acquired. The importance of more thorough teaching for young medical women than is given to pupil midwives in existing hospitals is obvious when it is remembered that the work of a doctor often begins where that of a midwife ends. This is especially the case in India, where European doctors, even women, are only sent for by the natives in circumstances of extreme difficulty or urgency." There is a great deal which is distinctive in connection with this maternity work at Clapham. What has been now mentioned is especially so, and one is hardly surprised to hear that Dr. McCall has as many as ninety pupils. Medical women's work in India is kept well in view, and a careful and special training in midwifery offered to students who think of going there.

In the matter of patients' fees, Dr. McCall and those acting with her aim at bringing about a radical reform. We are accustomed now to hospitals even in poor districts where pay-patients are the exception, not the rule, but this is an hospital for a poor district where free patients are the exception, not the rule. If the poor become pay-patients, the classes above them will surely be pay-patients also, and we shall be finding a solution of the question of maintaining the hospitals. The "hospital letter" system is pointedly discouraged. "No letters required" is the brief direction upon the printed cards circulated amongst the poor, and it is often the same class of persons who elsewhere beg letters from subscribers who here pay 5s. a week for themselves. Letters, of course, mean that the rich pay for the poor, a good thing when there is real necessity for it, but surely a bad thing when it only pauperises those who could pay for themselves. We hope Dr. McCall may not lead us to discard the letter system entirely, but for the sake of the hospitals, and for the sake of the people themselves, it will be well if she teaches us to be more judicious in using it. The fees, however, do not wholly meet the necessary expenses. They are small fees, and even women physicians experience what we mean by "bad debts." The out-patient maternity and the dispensary are nearly self-supporting, and we expect an encouraging report of the hospital as it becomes better known. But the preliminary cost of such an undertaking is considerable, and it is also desired to have a few free beds. Mrs. Ritchie appeals for small annual subscriptions to meet these expenses.

The Clapham Maternity Hospital offers co-operation with those engaged in rescue work. We could expect no less from an institution wholly controlled by women. The contempt of her own sex is one of the heaviest penalties incurred by a woman who falls from purity, and is often the most potent deterrent. Still, we could not imagine any woman saying of another: "As she has so far fallen let her fall entirely." This is really the judgment upon those young girls who go astray whilst in respectable service, and are then consigned to the companionship of about the worst class of the inmates of a workhouse. Girls like those described are received at the Clapham Maternity Hospital at 10s. a week, and those engaged in rescue work are glad to have the privilege of sending such cases. Trained nurses are employed as required, and the general nursing seems to have been undertaken by women of the Bible and Domestic Female Mission, who receive regular instruction, as we understand, from Dr. McCall.

The hospital is situated in a large and populous district, and at a considerable distance from any other maternity institution. If it be a condition of success in such an undertaking to offer peculiar advantages of its own, both to the profession and to the public, that condition is not wanting in this case. What we see, so far, is the successful effort o women physicians to strike out a special work of their own. Many who strongly opposed the admission of women to the profession were still ready to admit their probable usefulness in maternity work, not only at home, but especially in India. The day is past for prescribing so limited an ambition to medical women, but, it may be observed that this particular prediction is finding fulfilment in the unpretentious and successful work which Dr. Annie McCall is directing at Clapham.

